# Direct and Indirect Effects of Food and Nutrition Security on Dietary Choice and Healthfulness of Food Choice: Causal Mediation Analysis

**DOI:** 10.1016/j.cdnut.2024.102081

**Published:** 2024-01-13

**Authors:** Jessica L Thomson, Alicia S Landry, Tameka I Walls

**Affiliations:** 1 Delta Human Nutrition Research Program, USDA Agricultural Research Service, Stoneville, MS, United States; 2 Department of Nutrition and Family Sciences, University of Central Arkansas, Conway, AR, United States

**Keywords:** food security, nutrition security, dietary choice, healthful food choice, limited availability, utilization barriers, causal mediation, directed acyclic graph

## Abstract

**Background:**

Links between diet and food security are well established, but less is known about how food and nutrition security affect a household’s ability to decide what to consume.

**Objectives:**

This study’s purpose was to quantify and compare causal pathways from *1*) food and nutrition security to perceived dietary choice and *2*) food and nutrition security to perceived healthfulness of food choice while testing for mediation by perceived limited availability of foods and utilization barriers to healthful meals.

**Methods:**

Causal mediation analysis was conducted using an observational data set. Exposures included food insecurity and nutrition insecurity; mediators included perceived limited availability and utilization barriers; outcomes included perceived dietary choice and healthfulness choice; covariates included income and education.

**Results:**

Dietary choice (range 0–4) was 0.9 to 1.1 points lower for participants with food/nutrition insecurity compared with participants with food/nutrition security (direct effects). Neither mediation nor moderation by perceived limited availability were present. Seventeen percent and 11 %, respectively, of the effects of food and nutrition security on dietary choice could be contributed to utilization barriers (mediation). Moderation by utilization barriers was present only for nutrition security (differences in dietary choice only present when barriers were low). Healthfulness choice (range 0–4) was 0.6 to 0.7 points lower for participants with food/nutrition insecurity compared with participants with food/nutrition security (direct effects). Mediation by perceived limited availability and utilization barriers was not present. Moderation was present only for nutrition security (differences in healthfulness choice only present when perceived limited availability was low; differences in healthfulness choice only present when barriers were low).

**Conclusions:**

Food and nutrition security affect food choices, with utilization barriers acting as an intermediary step. When environmental and household utilization barriers to healthful food purchasing and preparation are high, the ability to decide what to consume does not differ between households with nutrition security and those with nutrition insecurity.

## Introduction

Food security has been defined as “access by all people at all times to enough food for an active, healthy life,” whereas food insecurity is defined as low or very low food security [1]. Food insecurity is characterized by a household’s limited ability to acquire adequate food due to lack of money and other resources, and it affects over 10 % of (13 million) households in the United States [2]. Food insecurity estimates are higher for households with children (13 %) and low income (27 %), and where the head of the household has less than a high school education (25 %) and is of Black race (20 %) or Hispanic ethnicity (16 %) [2]. Further, households with food insecurity are disproportionately affected by diet-related diseases such as diabetes and cardiovascular disease [3], likely due to the lower diet quality found in individuals (both adults and children) with food insecurity [4]. Although links between dietary intake and food security are well established [4,5], less is known about how food security affects a household’s ability to decide what to consume. Determining how food security affects food choices, particularly healthful food choices such as fruits and vegetables, may be helpful when conducting needs assessments in populations of interest prior to implementing an intervention, determining effectiveness of policies and programs designed to improve the nation’s dietary intake, and when analyzing public health surveillance data.

Food choices are influenced by both individual-level characteristics, such as socioeconomic status and food security, and by broader environmental factors, such as food systems and government policies [6]. One critical and frequently measured component of food systems is geographic food access or the ability to find food retail within a community [7]. Although policies have been developed to intervene upon geographic food access as a means to reduce the prevalence of food insecurity within a community, findings regarding the association between geographic food access and food insecurity have been inconsistent [8]. More consistent associations have been found among an individual’s perception of their food environment, food security, and food that is purchased and consumed [[9], [10], [11]]. Individuals may be less likely to buy healthier food options, such as fruits and vegetables, if they perceive their food environment to be poor [9] thus negatively affecting their diet quality. In a study conducted across multiple communities in the rural Mississippi Delta, almost half of the participants reported that fruits and vegetables were not available at nearby stores, and over half reported that they cannot keep fruits and vegetables fresh at home [12]. Additionally, approximately one-third of participants reported difficult preparation and taste as barriers to fruit and vegetable consumption [12]. Taken together, these results suggest that low perceived availability of healthful foods as well as preparation barriers may play important roles in an individual’s food choices. However, whether factors such as food availability and food preparation barriers can help explain the mechanistic action of food security on food choice has yet to be determined.

Whereas the focus of food security is on the ability to acquire enough food, nutrition security, a relatively new concept that does not have a single, universally accepted definition, focuses on the ability to acquire food that promotes health. For the purposes of the current study, the definition of nutrition security as “having consistent access, availability, and affordability of foods and beverages that promote well-being and prevent (and if needed, treat) disease” was used [13]. Nutrition security, distinct from food security, is viewed by some researchers as encompassing food security [14] and the next step in addressing the nation’s disparities in nutrition quality and corresponding diet-related diseases [13]. However, the shift in focus from food to nutrition security requires careful evaluation of appropriate screening tools alongside conventional measures of food security [13]. Additionally, similarities and differences between the 2 concepts in terms of associations with food choice are not well established in the literature and require further elucidation on their mechanistic action on food choice. In this study, it is hypothesized that perceived availability of foods and perceived barriers to meal preparation are intermediaries on the pathways between food and nutrition security and food choice. Thus, the purpose of the study was to conduct a secondary analysis of an existing observational data set to quantify and compare causal pathways from *1*) food and nutrition security to dietary choice and *2*) food and nutrition security to healthfulness of food choice while also testing for potential mediation by utilization barriers to healthful meals and perceived limited availability of foods in the pathways.

## Methods

### Design and sample

The original data were collected by researchers at the Gretchen Swanson Center for Nutrition (GSCFN) with the purpose of identifying food insecurity related measurement gaps and developing measures to address these gaps [15]. In particular, GSCFN researchers were interested in a broader view of food insecurity that encompasses nutritional adequacy of and personal choice in foods (e.g., identifying households who can afford food but may still have external limitations on their ability to acquire foods that meet their health needs and food preferences) [15]. With the help of partner organizations (*n* = 7) that worked with households at risk of or experiencing food insecurity, individuals were recruited across 5 states to pilot test measures that were developed [15]. Recruitment occurred from April to June 2021 and was conducted via email, text, and/or flyer. Inclusion criteria included ≥18 y of age, understood English, could answer questions about themselves and their household, and from a household experiencing food insecurity or at risk of food insecurity [15]. Pilot surveys were either web-based or paper and contained ∼75 to 85 items (depending on skip patterns), with 13 items representing the new measures. Each household completed a single survey, and participants received a gift card worth 25 US dollars (USD) for survey completion. The study was reviewed and approved by the University of Nebraska Medical Center Institutional Review Board (231-20-EX). All survey participants provided written informed consent. Complete details about the original study may be found elsewhere [15].

### Measures

Household food security was measured using the US Food Security Survey Module that comprises 10 questions for households without children <18 y of age and 18 questions for households with children [16]. Household food security was classified as full (no affirmative responses to questions), marginal (1–2 affirmative responses), low (3–5 and 3–7 affirmative responses for households without and with children, respectively), and very low (6–10 and 8–18 affirmative responses for households without and with children, respectively). For the purposes of the current study, food security was defined as full or marginal household food security, and food insecurity was defined as low or very low household food security.

Household nutrition security was measured based on a 12-mo time frame using a scale comprising 4 questions asking about a household’s need to eat foods not good for health because they could not get other types of foods and because they could not get healthful food, worry that food they were eating would hurt their health, and need to eat the same food several days in a row due to lack of money. Specific question wording may be found on the GSCFN website [17]. Response options included never, rarely, sometimes, often, or always and were scored from 0 to 4 points. Scale scores were calculated as means of the item scores, with mean scores ≤2 classified as low nutrition security or nutrition insecurity and mean scores >2 classified as nutrition security [15]. A sixth response option, don’t know, was not scored and treated as missing in the current analysis. The development, validation, and utility of the nutrition security measure may be found elsewhere [15].

Perceived dietary choice was measured using a scale comprising 3 questions asking about a household’s ability to decide what they eat. Specifically, questions addressed a household’s need to eat foods they did not want because they could not get other types of foods, eat food that was always changing because they did not know what they would be able to eat, and lack of control over the food they ate. Perceived healthfulness choice also was measured using a scale comprising 3 questions asking about a household’s control over consumption of quality fruits and vegetables, foods that were good for health, and processed foods (i.e., from a box, bag, or can). Specific examples of processed foods included mac and cheese, ramen noodles, canned ravioli, and frozen TV dinners. All questions were phrased within a 12-mo timeframe. Response options included never, rarely, sometimes, often, or always and were scored from 0 to 4 points. Scale scores were calculated as means of the item scores, with higher scores indicating greater choice [15]. A sixth response option, don’t know, was not scored and treated as missing in the current analysis. Higher dietary choice scores were associated with lower odds of fair/poor general health while higher healthfulness choice scores were associated with lower odds of low scratch-cooked meal intake and high fast food meal intake [15]. Additional details about the development, validation, and utility of the dietary and healthfulness choice measures may be found elsewhere [15].

Perceived limited availability was measured using a scale comprising 8 questions split into 2 sections. The first section asked about the types of food outlets at which a household got food and included 8 types (e.g., grocery store, big box store, convenience store, farmers’ market). This question was followed by opinion questions about food that was available at the food outlets selected (very few quality fruits and vegetables, liked foods, and foods good for health). The second section asked about the types of food sources from which a household got food and included 4 sources (e.g., food banks, donations, grown, discarded). This question again was followed by the same 3 opinion questions as the first section. Only the first section was used in the current study. Utilization barriers were measured using a scale comprising 8 questions asking about a household’s cooking skills (food selection or options, meal preparation, time) and storage/cooking equipment (e.g., access to cold storage, appliances, utensils, sanitation). All questions were phrased within a 12-mo timeframe. Specific question wording may be found on the GSCFN website [18]. Response options included never true, sometimes true, or often true and were scored as 0 (never) or 1 point (sometimes and often). Scale scores were calculated as sums of the item scores. Perceived limited availability was categorized as none (0 points), low (1 point), moderate (2 points), and high (3 points) [19]. For analytic purposes, perceived limited availability was categorized as low (0–1 points) and high (2–3 points). Utilization barriers scores ranged from 0 to 8 points, with higher scores indicating more barriers. A sixth response option, don’t know, was not scored and treated as missing in the current analysis. The development, validation, and utility of the perceived limited availability and utilization barriers measures may be found elsewhere [19].

Sociodemographic characteristics of interest included age (y), sex (male or female), race/ethnicity, education, employment, annual income (in USD), number of adults and children (<18 y of age) in the household, and recipient of US government food assistance programs [Supplemental Nutrition Assistance Program (SNAP) or Special Supplemental Nutrition Program for Women, Infants, and Children (WIC)]. Race/ethnicity categories included American Indian/Alaskan Native, Asian/Asian American, Black/African American, Hispanic/Latino(a), Middle Eastern/North African, Native Hawaiian/Pacific Islander, White/European American, and multiracial/multiethnic (including race/ethnicity not listed). Education categories included less than high school, high school diploma/General Educational Development test (GED), some college, associate degree/trade school/professional certificate, bachelor’s degree, and medical/law/or graduate school. For analytic purposes, education categories were combined as less than or equal to high school diploma/GED, some college/associate degree, and greater than or equal to bachelor’s degree. Employment categories included not working (retired, disabled, homemaker, full-time student), working <30 h/wk, and working ≥30 h/wk. Annual income was computed by multiplying the median of the range (selected from 14 discrete ranges) by 12. The number of adults in the household was categorized as 1, 2 or ≥3, and the number of children was categorized as 0, 1, 2, and ≥3.

### Statistical analyses

Statistical analyses were conducted using SAS version 9.4 (SAS Institute, Inc.), with significance set at *P* ≤ 0.05. Descriptive statistical methods (frequencies, percentages, means, and SD) were used to summarize participant characteristics. Comparisons between participants classified by food and nutrition security status (insecurity compared with security) were performed using chi-square tests of association or Fisher’s exact test for categorical variables and 2-sample *t* tests (approximately normal distribution) or Wilcoxon rank sum 2-sample tests (skewed distribution) for ordinal and continuous variables. Distributions of ordinal and continuous variables were visually inspected for approximate normality.

**FIGURE 1:**
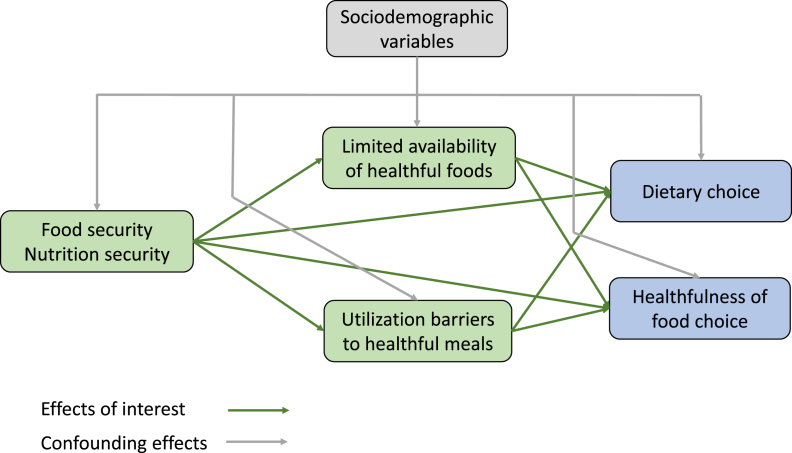
Causal model for direct and indirect effects of food and nutrition security on dietary choice and healthfulness of food choice mediated by perceived limited availability of healthful foods and utilization barriers to healthful meals.

Prior to conducting causal mediation analysis, a directed acyclic graph (DAG) was created and used to guide the analysis (Figure 1). A DAG is a graphical representation of causal effects between variables that can help to determine whether bias is reduced or increased when conditioning on covariates [20]. Causation is indicated by an arrow connecting 2 variables whereas variables with no direct causal association are left unconnected. For the purposes of the current study, causation is defined in counterfactual terms—had the exposure (security) differed, the outcome would have differed (perceived choice) [20]. As can be seen in the DAG, direct effects of food and nutrition security on dietary choice and healthfulness of food choice (healthfulness choice) as well as indirect effects mediated through limited availability of healthful foods (perceived limited availability) and utilization barriers to healthful meals (utilization barriers) were hypothesized. To guide the selection of variables to include in the model, the CausalGraph procedure was used to identify adjustment sets of variables that can be used to remove or block spurious or confounding associations between the treatment/exposure and outcome variables. The initial theoretical set of sociodemographic variables included age, sex, income, race, education, and employment status. Based on the data (e.g., bivariate associations between sociodemographic variables and outcome variables), a smaller empirical set of sociodemographic variables was created and included age, race, and income. The backdoor adjustment criterion was used to identify the causal effect based on observed variables only [21]. The minimal valid covariate adjustment sets included 3 variables—annual income, education, and food security. Because perceived limited availability and utilization barriers were of particular interest, the valid adjustment sets containing them and the minimal adjustment set variables were selected for modeling.

To estimate causal mediation effects from the observational data set, the CausalMed procedure was used. This procedure uses generalized linear models for describing relationships among outcome, treatment/exposure, and mediator variables while allowing for the inclusion of covariates and dealing with confounding by implementing the regression approach of VanderWeele [22]. Modeling results include estimates of main causal mediation effects—total effect, controlled direct effect, natural direct effect, and natural indirect (mediation) effect—and general linearized models for the outcome variable and the mediator variable using maximum likelihood estimation. Because the CausalMed procedure only allows for single treatment, outcome, and mediator variable, 8 models were tested and can be found in Table 1. Exposure variables included food insecurity and nutrition insecurity (binary); mediator variables included perceived limited availability (binary) and utilization barriers (continuous); outcome variables included dietary choice and healthfulness choice (continuous); and covariates included annual income (standardized continuous), education (categorical), and the other mediator variable (perceived limited availability or utilization barriers). Although distributions of the outcome variables did not appear approximately normal, linear regression methods were robust against nonnormality [23].

**TABLE 1:** Causal mediation models tested

Model	Exposure	Mediator	Outcome	Covariates
1	Food insecurity	PLA	Dietary choice	UB, annual income, education
2	Nutrition insecurity	PLA	Dietary choice	UB, annual income, education
3	Food insecurity	UB	Dietary choice	PLA, annual income, education
4	Nutrition insecurity	UB	Dietary choice	PLA, annual income, education
5	Food insecurity	PLA	Healthfulness choice	UB, annual income, education
6	Nutrition insecurity	PLA	Healthfulness choice	UB, annual income, education
7	Food insecurity	UB	Healthfulness choice	PLA, annual income, education
8	Nutrition insecurity	UB	Healthfulness choice	PLA, annual income, education

Abbreviations: PLA, perceived limited availability; UB, utilization barriers.

## Results

Of the 380 participants in the original data set, 308 (81 %) and 301 (79 %) had complete food security and nutrition security data, respectively. Characteristics of the current analytic sample by food security status are presented in Table 2. Comparisons between participants with food insecurity and with food security revealed several significant differences. Participants with food insecurity were more likely to have some college experience (45 % compared with 29 %) and less likely to have a bachelor’s degree or higher (18 % compared with 32 %) than participants with food security. Participants with food insecurity also were more likely to receive SNAP (68 % compared with 54 %), have nutrition insecurity (51 % compared with 5 %), and have high perceived limited availability than participants with food security (44 % compared with 24 %). Additionally, on average, participants with food insecurity were younger (43 compared with 48 years), had lower annual income (15,202 compared with 21,399 USD), and scored lower for dietary choice (2.2 compared with 3.5) and healthfulness choice (2.3 compared with 3.1) but higher for utilization barriers (2.8 compared with 0.7) than participants with food security.

**TABLE 2:** Selected participant characteristics for analytic sample by food and nutrition security status

Characteristic	*n*	Food insecurity		Food security	*P*^1^	Nutrition insecurity		Nutrition security		*P*^1^
		(*N* = 212)		(*N* = 96)		(*N* = 110)		(*N* = 191)		
		%	*n*	%		*n*	%	*n*	%	
Adults (household)					0.054					0.086
1	76	36.2	28	29.2		41	38.0	62	32.5	
2	81	38.6	51	53.1		37	34.3	90	47.1	
≥3	53	25.2	17	17.7		30	27.8	39	20.4	
Children (household <18 y of age)^2^					0.148					0.194
0	77	36.3	48	50.0		39	35.5	84	44.0	
1	32	15.1	11	11.5		14	12.7	28	14.7	
2	29	13.7	12	12.5		20	18.2	20	10.5	
≥3	74	34.9	25	26.0		37	33.6	59	30.9	
Sex					0.154					0.916
Male	38	18.4	24	25.5		22	20.8	40	21.3	
Female	169	81.6	70	74.5		84	79.2	148	78.7	
Race/ethnicity					3					3
American Indian/Alaskan Native	3	1.5	0	0.0		2	1.9	1	0.5	
Asian/Asian American	5	2.4	8	8.7		1	1.0	12	6.5	
Black/African American	37	18.0	16	17.4		26	24.8	25	13.4	
Hispanic/Latino(a)	49	23.8	17	18.5		19	18.1	45	24.2	
Middle Eastern/North African	3	1.5	0	0.0		2	1.9	1	0.5	
Native Hawaiian/Pacific Islander	0	0.0	0	0.0		0	0.0	0	0.0	
White/European American	92	44.7	49	53.3		46	43.8	92	49.5	
Multiracial/multiethnic^4^	17	8.3	2	2.2		9	8.6	10	5.4	
Education					0.009					0.050
≤High school diploma/GED	75	36.4	36	39.6		36	34.0	71	38.6	
Some college/associate degree^5^	93	45.1	26	28.6		52	49.1	65	35.3	
≥Bachelor’s degree	38	18.4	29	31.9		18	17.0	48	26.1	
Employment					0.569					0.836
Not working^6^	126	61.2	60	64.5		69	64.5	114	61.6	
Working <30 h/wk	44	21.4	15	16.1		19	17.8	38	20.5	
Working ≥30 h/wk	36	17.5	18	19.4		19	17.8	33	17.8	
SNAP recipient					0.025					0.103
No	69	32.5	44	45.8		34	30.9	77	40.3	
Yes	143	67.5	52	54.2		76	69.1	114	59.7	
WIC recipient					0.506					0.024
No	177	83.5	83	86.5		86	78.2	168	88.0	
Yes	35	16.5	13	13.5		24	21.8	23	12.0	
Food security^7^					<0.001					<0.001
Very low	125	59.0	0	0.0		78	70.9	44	23.0	
Low	87	41.0	0	0.0		27	24.5	59	30.9	
Marginal	0	0.0	41	42.7		5	4.5	34	17.8	
Full	0	0.0	55	57.3		0	0.0	54	28.3	
Nutrition security^8^					<0.001					<0.001
No (score ≤2)	105	50.5	5	5.4		110	100.0	0	0.0	
Yes (score >2)	103	49.5	88	94.6		0	0.0	191	100.0	
Perceived limited availability					<0.001					<0.001
None	36	17.0	43	44.8		7	6.4	71	37.2	
Low	42	19.8	14	14.6		16	14.5	38	19.9	
Moderate	40	18.9	16	16.7		22	20.0	32	16.8	
High	94	44.3	23	24.0		65	59.1	50	26.2	
	Mean	SD	Mean	SD	*P*^9^	Mean	SD	Mean	SD	*P*^9^
Age (y)	43.3	13.4	48.0	15.4	0.007	43.4	12.6	45.6	15.0	0.172
Annual income (USD)	15,202	10,221	21,399	14,497	<0.001	14,271	8906	18,559	13,219	0.001
Dietary choice^10^	2.2	0.8	3.5	0.6	<0.001	1.9	0.7	3.0	0.8	<0.001
Healthfulness choice^10^	2.3	0.8	3.1	1.0	<0.001	2.1	0.6	2.8	1.0	<0.001
Nutrition security^11^	2.2	0.7	3.4	0.6	<0.001	1.7	0.4	3.1	0.6	<0.001
Utilization barriers^12^	2.8	2.3	0.7	1.3	<0.001	3.6	2.4	1.3	1.7	<0.001

Abbreviations: GED, general educational development test; SNAP, Supplemental Nutrition Assistance Program; USD, United States dollar; WIC, Special Supplemental Nutrition Program for Women, Infants, and Children.

1 *P* value for chi-square tests or Fisher’s exact test.

2 Includes children <18 y of age who are not under legal guardianship.

3 Chi-square test invalid due to empty/small cell sizes.

4 Includes another race/ethnicity not listed.

5 Includes trade school or professional certificate.

6 Includes retired, disabled, homemaker, or full-time student.

7 Food insecurity = low and very low; food security = full or marginal.

8 Nutrition insecurity = score ≤2; nutrition security = score >2 (range 0–4 points).

9 *P* value for 2-sample *t* test or Wilcoxon rank sum 2-sample test.

10 Higher values indicate greater choice (range 0–4 points).

11 Higher values indicate higher security.

12 Higher values indicate more perceived barriers (range 0–8 points).

Characteristics of the current analytic sample by nutrition security status are also presented in Table 2. Comparisons between participants with nutrition insecurity and with nutrition security revealed several significant differences. Participants with nutrition insecurity were more likely to have some college experience (49 % compared with 35 %) and less likely to have a bachelor’s degree or higher (17 % compared with 26 %) than participants with nutrition security. Participants with nutrition insecurity also were more likely to receive WIC (22 % compared with 12 %), have food insecurity (95 % compared with 54 %), and have high perceived limited availability (59 % compared with 26 %) than participants with nutrition security. Additionally, on average, participants with nutrition insecurity had lower annual income (14,271 compared with 18,559 USD) and scored lower for dietary choice (1.9 compared with 3.0) and healthfulness choice (2.1 compared with 2.8) but higher for utilization barriers (3.6 compared with 1.3) than participants with nutrition security.

### Causal mediation analysis – dietary choice

Results from the models testing for mediation of the causal pathways between food and nutrition security and dietary choice by perceived limited availability are presented in Table 3. For the food security causal pathway, dietary choice was 1.0 points lower for participants with food insecurity than participants with food security (total and natural direct effects). The controlled direct effects of low and high perceived limited availability on dietary choice were of similar magnitude, indicating an interaction effect was not present. The natural indirect (mediation) effect also was not significant. For the nutrition security causal pathway, dietary choice was 0.9 points lower for participants with nutrition insecurity than participants with nutrition security (total and natural direct effects). The controlled direct effects of low and high perceived limited availability on dietary choice were of similar magnitude, indicating an interaction effect was not present. The natural indirect (mediation) effect also was not significant.

**TABLE 3:** Causal mediation analysis for effects of food and nutrition security on dietary choice^1^

Component	Food security				Nutrition security			
	Mediator = perceived limited availability							
	Estimate	95 % Wald CI		P	Estimate	95 % Wald CI		P
Total effect	−1.0	−1.2	−9.9	<0.001	−0.9	−1.1	−0.7	<0.001
Controlled direct effect (low)^2^	−0.9	−1.1	−7.2	<0.001	−1.1	−1.4	−0.8	<0.001
Controlled direct effect (high)^2^	−1.0	−1.3	−7.5	<0.001	−0.7	−1.0	−0.5	<0.001
Natural direct effect^3^	−1.0	−1.1	−9.9	<0.001	−0.9	−1.1	−0.7	<0.001
Natural indirect effect^4^	0.0	0.0	−0.7	0.516	0.0	0.0	0.1	0.289
% Mediated	1.2	−2.4	0.7	0.513	−5.1	−14.6	4.4	0.294
% Interaction	7.9	−13.1	0.7	0.462	10.6	−1.3	22.4	0.081
	Mediator = utilization barriers							
Total effect	−1.1	−1.3	−0.9	<0.001	−1.1	−1.3	−0.9	<0.001
Controlled direct effect (mean)^2^	−0.9	−1.1	−0.7	<0.001	−0.9	−1.1	−0.7	<0.001
Controlled direct effect (−1 SD)^2^	−1.0	−1.2	−0.8	<0.001	−1.1	−1.4	−0.8	<0.001
Controlled direct effect (+1 SD)^2^	−0.8	−1.2	−0.4	<0.001	−0.7	−0.9	−0.4	<0.001
Natural direct effect^3^	−0.9	−1.1	−0.8	<0.001	−1.0	−1.2	−0.8	<0.001
Natural indirect effect^4^	−0.2	−0.3	−0.1	<0.001	−0.1	−0.2	0.0	0.030
% Mediated	16.9	9.2	24.6	<0.001	10.5	1.0	20.0	0.031
% Interaction	−1.8	−6.8	3.3	0.495	−10.2	−19.1	−1.3	0.025

Abbreviation: CI, confidence interval.

1 Exposure = food/nutrition insecurity; control = food/nutrition security; covariates included education, annual income, and utilization barriers/perceived limited availability when not acting as mediator.

2 Food/nutrition security effect not due to interaction or mediation.

3 Food/nutrition security direct effect including interaction.

4 Food/nutrition security indirect (mediated) effect including interaction.

Results from the models testing for mediation of the causal pathways between food and nutrition security and dietary choice by utilization barriers are also presented in Table 3. For the food security causal pathway, dietary choice was 1.1 points lower for participants with food insecurity than participants with food security (total effect). The effects of utilization barriers on dietary choice were of similar magnitude at different values, indicating an interaction effect was not present. Mediation was present, indicating that 17 % of the effect of food security on dietary choice could be contributed to utilization barriers. For the nutrition security causal pathway, dietary choice was 1.1 points lower for participants with nutrition insecurity than participants with nutrition security (total effect). The effects of utilization barriers on dietary choice varied at different values (larger magnitude for lower value than higher value), indicating an interaction effect was present. Mediation was also present, indicating 11 % of the effect of nutrition security on dietary choice can be contributed to utilization barriers.

### Multivariable linear regression models – dietary choice

Presented in Table 4 are results from the multivariate linear regression models testing for significant explanatory variables of dietary choice. For the perceived limited availability model, utilization barriers and food security were significant explanatory variables. A 1-point increase in utilization barriers resulted in a 0.1-point decrease in dietary choice. Dietary choice was 1.0 points lower for participants with food insecurity than participants with food security. Results were remarkably similar for the model replacing food security with nutrition security (i.e., significant explanatory variables and magnitude of effects).

**TABLE 4:** Multivariate linear regression models for dietary choice

Parameter	Food security				Nutrition security			
	Mediator = perceived limited availability							
	Estimate	95 % Wald CI		*P*	Estimate	95 % Wald CI		*P*
Intercept	3.5	3.2	3.7	<0.001	3.3	3.1	3.5	<0.001
≤High school education^1^	0.0	−0.2	0.2	0.782	−0.1	−0.3	0.1	0.529
Some college education^1^^,^^2^	0.1	−0.1	0.3	0.410	0.0	−0.2	0.2	0.907
Annual income^3^	0.0	−0.1	0.1	0.565	0.1	0.0	0.2	0.087
Utilization barriers	−0.1	−0.2	−0.1	<0.001	−0.1	−0.2	−0.1	<0.001
FI vs FS/NI vs NS	−1.0	−1.3	−0.7	<0.001	−1.1	−1.4	−0.8	<0.001
High vs low PLA	0.1	−0.2	0.4	0.462	−0.2	−0.4	0.0	0.081
Interaction^4^	0.1	−0.2	0.5	0.468	0.4	0.0	0.7	0.059
	Mediator = utilization barriers							
Intercept	3.4	3.2	3.7	<0.001	3.2	3.0	3.5	<.001
≤High school education^1^	0.0	−0.2	0.2	0.769	−0.1	−0.3	0.1	0.336
Some college education^1^^,^^2^	0.1	−0.1	0.3	0.486	0.0	−0.2	0.2	0.956
Annual income^3^	0.0	−0.1	0.1	0.580	0.1	0.0	0.2	0.088
Low vs high PLA	0.2	0.0	0.4	0.028	0.1	−0.1	0.3	0.374
FI vs FS /NI vs NS	−1.0	−1.2	−0.8	<0.001	−1.1	−1.4	−0.8	<0.001
Utilization barriers	−0.2	−0.3	−0.1	0.004	−0.2	−0.2	−0.1	<0.001
Interaction^4^	0.0	−0.1	0.1	0.484	0.1	0.0	0.2	0.014

Abbreviations: CI, confidence interval; FI, food insecurity; FS, food security; NI, nutrition insecurity; NS, nutrition security; PLA, perceived limited availability.

1 Reference category is less than or equal to bachelor’s degree.

2 Includes associate degree, trade school, or professional certificate.

3 Annual income is standardized (1 standard deviation = 12,043 United States dollars).

4 Interaction between food/nutrition security and mediator variable.

For the utilization barriers model, perceived limited availability, food security, and utilization barriers were significant explanatory variables of dietary choice. Dietary choice was 0.2 points higher for participants with low perceived limited availability than participants with high perceived limited availability. Additionally, dietary choice was 1.0 points lower for participants with food insecurity than participants with food security. Finally, a 1-point increase in utilization barriers resulted in a 0.2-point decrease in dietary choice. Results were somewhat similar for the model replacing food security with nutrition security. Nutrition security, utilization barriers, and the interaction between nutrition security and utilization barriers were significant explanatory variables for dietary choice. Dietary choice was 1.1 points lower for participants with nutrition insecurity than participants with nutrition security. A 1-point increase in utilization barriers resulted in 0.2- and 0.1-point decreases in dietary choice for individuals with nutrition security and those with nutrition insecurity, respectively. Results from the mediator models are not discussed but are found in Supplemental Table 1.

### Causal mediation analysis – healthfulness choice

Results from the models testing for mediation of the causal pathways between food and nutrition security and healthfulness choice by perceived limited availability are presented in Table 5. For the food security causal pathway, healthfulness choice was 0.6 points lower for participants with food insecurity than participants with food security (total and natural direct effects). The controlled direct effects of low and high perceived limited availability on healthfulness choice were of similar magnitude, indicating an interaction effect was not present. The natural indirect (mediation) effect also was not significant. For the nutrition security causal pathway, healthfulness choice was 0.6 points lower for participants with nutrition insecurity than participants with nutrition security (total and natural direct effects). The controlled direct effects of low and high perceived limited availability on healthfulness choice differed (significant at low value and nonsignificant at high value), indicating an interaction effect was present. The natural indirect (mediation) effect was not significant.

**TABLE 5:** Causal mediation analysis for effects of food and nutrition security on healthfulness choice^1^

Component	Food security				Nutrition security			
	Mediator = perceived limited availability							
	Estimate	95 % Wald CI		*P*	Estimate	95 % Wald CI		*P*
Total effect	−0.6	−0.8	−0.4	<0.001	−0.6	−0.8	−0.3	<0.001
Controlled direct effect (low)^2^	−0.7	−1.0	−0.4	<0.001	−1.0	−1.3	−0.6	<0.001
Controlled direct effect (high)^2^	−0.5	−0.8	−0.2	0.002	−0.2	−0.5	0.0	0.095
Natural direct effect^3^	−0.6	−0.8	−0.4	<0.001	−0.6	−0.8	−0.4	<0.001
Natural indirect effect^4^	0.0	−0.1	0.0	0.507	0.0	−0.1	0.1	0.587
% Mediated	3.2	−5.9	12.4	0.489	−4.5	−20.9	11.9	0.590
% Interaction	−24.4	−67.0	18.1	0.261	−96.5	−166	−26.6	0.007
	Mediator = utilization barriers							
Total effect	−0.7	−0.9	−0.5	<0.001	−0.6	−0.8	−0.4	<0.001
Controlled direct effect (mean)^2^	−0.6	−0.8	−0.3	<0.001	−0.5	−0.7	−0.3	<0.001
Controlled direct effect (−1 SD)^2^	−0.6	−0.9	−0.4	<0.001	−0.9	−1.2	−0.6	<0.001
Controlled direct effect (+1 SD)^2^	−0.5	−1.0	0.0	0.057	−0.1	−0.4	0.2	0.500
Natural direct effect^3^	−0.6	−0.8	−0.4	<0.001	−0.6	−0.9	−0.4	<0.001
Natural indirect effect^4^	−0.1	−0.2	0.0	0.072	0.1	−0.1	0.2	0.323
% Mediated	11.3	−1.2	23.8	0.076	−9.7	−29.2	9.8	0.329
% Interaction	−2.8	−12.6	6.9	0.568	−35.7	−60.2	−11.1	0.004

Abbreviation: CI, confidence interval.

1 Exposure = food/nutrition insecurity; control = food/nutrition security; covariates included education, annual income, and utilization barriers/perceived limited availability when not acting as mediator.

2 Food/nutrition security effect not due to interaction or mediation.

3 Food/nutrition security direct effect including interaction.

4 Food/nutrition security indirect (mediated) effect including interaction.

Results from the models testing for mediation of the causal pathways between food and nutrition security and healthfulness choice by utilization barriers are also presented in Table 5. For the food security causal pathway, healthfulness choice was 0.7 points lower for participants with food insecurity than participants with food security (total and natural direct effects). The controlled direct effects of utilization barriers on healthfulness choice were of similar magnitude, indicating an interaction effect was not present. The natural indirect (mediation) effect was also not significant. For the nutrition security causal pathway, healthfulness choice was 0.6 points lower for participants with nutrition insecurity than participants with nutrition security (total and natural direct effects). The controlled direct effects of utilization barriers on healthfulness choice varied at different values [higher at lower and mean values and lower (nonsignificant) at higher values] indicating an interaction effect was present. The natural indirect (mediation) effect was not significant.

### Multivariable linear regression models – healthfulness choice

Presented in Table 6 are results from the multivariate linear regression models testing for significant explanatory variables of healthfulness choice. For the perceived limited availability model, education, utilization barriers, food security, and perceived limited availability were significant explanatory variables. Healthfulness choice was 0.3 points lower for participants with less than or equal to a high school education than participants with greater than or equal to a bachelor’s degree. A 1-point increase in utilization barriers resulted in a 0.1-point decrease in healthfulness choice. Healthfulness choice was 0.7 points lower for participants with food insecurity than participants with food security and 0.6 points lower for participants with high than those with low perceived limited availability. Results were remarkably similar for the model replacing food security with nutrition security (i.e., significant explanatory variables and magnitude of effects) with the following exception. In the presence of low perceived limited availability, healthfulness choice was 1.0 points lower for participants with nutrition insecurity than participants with nutrition security, although healthfulness choice did not differ by nutrition security status in the presence of high perceived limited availability.

**TABLE 6:** Multivariate linear regression models for healthfulness choice

Parameter	Food security				Nutrition security			
	Mediator = perceived limited availability							
	Estimate	95 % Wald CI		*P*	Estimate	95 % Wald CI		*P*
Intercept	3.5	3.3	3.8	<0.0001	3.3	3.1	3.5	<0.0001
≤High school education^1^	−0.3	−0.5	0.0	0.027	−0.3	−0.5	0.0	0.023
Some college education^1^^,^^2^	0.0	−0.2	0.2	0.966	0.0	−0.3	0.2	0.690
Annual income^3^	0.0	−0.1	0.1	0.423	0.1	0.0	0.2	0.147
Utilization barriers	−0.1	−0.1	0.0	0.020	−0.1	−0.1	0.0	0.008
FI vs FS/NI vs NS	−0.7	−1.0	−0.4	<0.0001	−1.0	−1.3	−0.6	<0.0001
High vs low PLA	−0.6	−1.0	−0.3	<0.001	−0.6	−0.9	−0.4	<0.0001
Interaction^4^	0.2	−0.2	0.6	0.226	0.7	0.3	1.2	0.001
	Mediator = utilization barriers							
Intercept	3.0	2.7	3.3	<.0001	2.9	2.7	3.2	<0.0001
≤High school education^1^	−0.3	−0.5	0.0	0.020	−0.4	−0.6	−0.1	0.004
Some college education^1^^,^^2^	0.0	−0.3	0.2	0.936	−0.1	−0.3	0.2	0.610
Annual income^3^	0.0	−0.1	0.1	0.434	0.1	0.0	0.2	0.152
Low vs high PLA	0.5	0.3	0.7	<0.0001	0.4	0.2	0.6	<0.0001
FI vs FS/NI vs NS	−0.6	−0.9	−0.4	<0.0001	−0.9	−1.2	−0.6	<0.0001
Utilization barriers	−0.1	−0.2	0.0	0.167	−0.2	−0.2	−0.1	<0.0001
Interaction^4^	0.0	−0.1	0.2	0.557	0.2	0.1	0.3	<0.0001

Abbreviations: CI, confidence interval; FI, food insecurity; FS, food security; NI, nutrition insecurity; NS, nutrition security; PLA, perceived limited availability.

1 Reference category is less than or equal to bachelor’s degree.

2 Includes associate degree, trade school, or professional certificate.

3 Annual income is standardized (1 SD = 12,043 US dollars).

4 Interaction between food/nutrition security and mediator variable.

For the utilization barriers model, education, perceived limited availability, and food security were significant explanatory variables. Healthfulness choice was 0.3 points lower for participants with less than or equal to high school education than participants with greater than or equal to a bachelor’s degree. Additionally, healthfulness choice was 0.5 points higher for participants with low than those with high perceived limited availability and 0.6 points lower for participants with food insecurity than participants with food security. For the model replacing food security with nutrition security, education, perceived limited availability, nutrition security, utilization barriers, and the interaction between nutrition security and utilization barriers were significant explanatory variables. Healthfulness choice was 0.4 points lower for participants with less than or equal to high school education than participants with greater than or equal to a bachelor’s degree and 0.4 points higher for participants with low compared with high perceived limited availability. At lower values of utilization barriers, healthfulness choice was ∼0.9 points lower for participants with nutrition insecurity as compared to participants with nutrition security. The effect lessened in magnitude as utilization barriers increased such that the difference in healthfulness choice between participants with nutrition insecurity and those with nutrition security was no longer significant. Results from the mediator models are not discussed but are found in Supplemental Table 1.

## Discussion

In this study, causal mediation analysis was used to test and compare causal pathways from food and nutrition security to dietary and healthfulness choice directly and indirectly (mediated) through perceived limited availability and utilization barriers using an observational data set. The results support direct effects of both food and nutrition security on dietary choice and indirect effects mediated by utilization barriers but not by perceived limited availability. Positive associations between food security and diet quality are well established in the literature [4,5]. Hence, associations between food security and dietary choice are logical because lack of financial resources (food insecurity) would affect an individual’s food purchasing choices. Associations between nutrition security and dietary choice are not as well established, likely because nutrition security is a fairly new concept. The current study provides evidence that, similar to food security, nutrition security is associated with dietary choice, presumably because food security increases the likelihood of nutrition security [4]. Households with constrained economic resources experiencing food insecurity are also likely to be experiencing nutrition insecurity due to difficulties obtaining nutritionally balanced foods [4]. Perhaps more interesting than the direct effects of food and nutrition security on dietary choice are the indirect effects. Evidence for mediation of relationships between food and nutrition security and dietary choice by utilization barriers to healthful meals may partly explain how or why food and nutrition security affect food choices. The results suggest that households with food or nutrition insecurity lack the tools or supplies necessary for preparing healthful meals. Thus, these households may choose not to purchase foods that require specialized cooking utensils or equipment and take longer to prepare, opting instead for convenient boxed or canned foods with fast and simple preparation methods. In contrast to utilization barriers, the lack of evidence supporting the mediation of relationships between food and nutrition security and dietary choices by perceived limited availability of foods is surprising. The small (nonsignificant) effect size of perceived limited availability on dietary choice may partly explain its absence in the pathways between food and nutrition security and food choices. Regardless, the results from the current study suggest that barriers such as lack of storage, equipment, and cooking knowledge explain how food and nutrition security affect food choices, more so than perceived limited availability of foods. Confirmation of these results in other populations is needed.

Similar to dietary choice, results support direct effects of food and nutrition security on healthfulness choice. Again, associations between food and nutrition security and healthfulness choice are logical because lack of financial resources (food and nutrition insecurity) would affect an individual’s food purchasing choices, particularly healthful foods that tend to be more expensive. Dissimilar to dietary choice, no evidence was found to support mediated effects by either perceived limited availability or utilization barriers on relationships between food and nutrition security and healthfulness choice. It is possible that coping strategies used by households with food or nutrition insecurity may negate indirect effects of perceived limited availability on healthfulness choice. For example, households with food or nutrition insecurity may use grocery store coupons, which are often less frequently available for healthful food options [24]. Thus, whether households with food or nutrition insecurity perceive their food environment as limited has little bearing on the healthfulness of their food choices because they have no intention of purchasing such foods. This argument also may hold for the lack of mediation effects by utilization barriers, although the findings deserve further study.

The presence of interaction (moderation) between nutrition security and the 2 potential mediator variables, perceived limited availability and utilization barriers, is intriguing. Discrepancies in dietary choice and healthfulness choice between households with nutrition security and those with nutrition insecurity disappear in the presence of high perceived limited availability and high utilization barriers. Thus, greater presence of environmental or household barriers to healthful food purchasing and preparation levels the playing field between households with nutrition security and those with nutrition insecurity in terms of food choice. Conversely, moderation of relationships between food security and dietary and healthfulness choice were not found in the present study, although moderation of food security relationships has been previously reported. In a study conducted in 2 communities with high poverty rates and low access to healthy food retailers, greater perception of healthful food availability was associated with lower diet quality scores in participants with food security [25]. However, the opposite was observed for participants with very low food security—greater perception of healthful food availability was associated with higher diet quality scores [25]. The authors hypothesized that the unexpected result in participants with food security may be related to their reported higher use of personal vehicles to travel to their primary food stores, likely located outside their neighborhoods perceived to have poor food environments [25]. Taken together, these results suggest that food security is strongly tied to household income. Even when healthful foods are available and barriers to healthful eating are low, households with food insecurity still are unable to make more healthful dietary choices. The results should be generalized cautiously because both study populations were majority low income.

The finding that annual household income was not a significant explanatory variable for dietary or healthfulness choice was surprising as associations between income and diet have been previously reported [26,27]. A possible explanation could be the purposeful sampling of populations at risk of or experiencing food insecurity, resulting in the current study sample being majority low income [15]. The finding that education was an explanatory variable for healthfulness choice but not dietary choice is intriguing and suggests that education plays a greater role in healthful food choices compared with food choices in general. Clearly, differential associations among sociodemographic characteristics and overall food choices compared with healthful food choices deserve further study.

Limitations of this study include use of a convenience sample, which restricts the generalizability of study findings. The relationships hypothesized in the DAG could be incorrect, although most of the direct relationships have been reported previously in the food security literature. The statistical procedure used for causal mediation only allows for single exposure and mediator variables thus limiting how relationships among these variables can be tested. Finally, all data are self-reported and thus subject to bias.

In conclusion, food and nutrition security affect food choices, with evidence supporting utilization barriers to healthful meals as an intermediary step in the pathway. Additionally, when environmental and household utilization barriers to healthful food purchasing and preparation are high, the ability to decide what is eaten does not differ between households with nutrition security and those with nutrition insecurity. Strategies and policies that remove household utilization barriers may help lessen the burdens of both food and nutrition insecurity for those at risk of or experiencing food insecurity.

## Acknowledgments

We thank Drs Leah Carpenter and Eric Calloway for providing access to the data.

### Author contributions

The authors’ responsibilities were as follows – JLT, ASL, TIW: designed research; JLT: analyzed data; JLT, ASL, TIW: interpreted results; JLT: wrote article; ASL, TIW: edited article; JLT: had primary responsibility for the final content; and all authors: read and approved the final manuscript.

### Conflict of interest

ASL reports financial support was provided by USDA Agricultural Research Service and a relationship with USDA Agricultural Research Service that includes: funding cooperative agreements. All other authors report no conflicts of interest.

### Funding

This work was supported by the US Department of Agriculture (USDA), Agricultural Research Service (Project 6001-51000-004-00D). The supporter had no involvement or restrictions regarding the study design; collection, analysis, and interpretation of data; writing of the report; or submission of the report for publication. The findings and conclusions in this publication are those of the authors and should not be construed to represent any official USDA or US government determination or policy. USDA is an equal opportunity provider and employer.

### Data availability

Data described in the manuscript, code book, and analytic code will be made available upon request pending approval by Dr Leah Carpenter, Associate Director of the Gretchen Swanson Center for Nutrition.

## Appendix A Supplementary data

Multimedia component1 Multimedia component1
