# Supplementary material for: Direct and Indirect Effects of Food and Nutrition Security on Dietary Choice and Healthfulness of Food Choice: Causal Mediation Analysis
Source: Curr Dev Nutr. 2024 Jan 13;8(2):102081. doi: 10.1016/j.cdnut.2024.102081 (PMC10847738; doi:10.1016/j.cdnut.2024.102081)
Supplement: Multimedia component1 [file mmc1.docx]

| **SUPPLEMENTAL TABLE 1** | | | | | | | | |
| --- | --- | --- | --- | --- | --- | --- | --- | --- |
| Multivariate linear regression models for mediator variables | | | | | | | | |
|  | Food security | | | | Nutrition security | | | |
|  | Outcome = high perceived limited availability | | | | | | | |
| Parameter | Estimate | 95% Wald CI | | P | Estimate | 95% Wald CI | | P |
| Intercept | 1.2 | 0.5 | 1.8 | <.001 | -1.2 | -1.9 | -0.6 | <.001 |
| ≤High school education^1^ | -0.6 | -1.3 | 0.1 | 0.109 | 0.7 | 0.0 | 1.4 | 0.067 |
| Some college education^1,2^ | -0.9 | -1.5 | -0.2 | 0.013 | 0.8 | 0.1 | 1.5 | 0.024 |
| Annual income^3^ | -0.1 | -0.4 | 0.2 | 0.457 | 0.1 | -0.2 | 0.4 | 0.466 |
| Utilization barriers | -0.4 | -0.5 | -0.2 | <.001 | 0.3 | 0.2 | 0.5 | <.001 |
| FI vs FS/NI vs NS | -0.2 | -0.8 | 0.4 | 0.495 | 1.1 | 0.5 | 1.7 | 0.001 |
|  | Outcome = utilization barriers | | | | | | | |
| Intercept | 1.7 | 1.1 | 2.4 | <.001 | 2.0 | 1.4 | 2.5 | <.001 |
| ≤High school education^1^ | 0.0 | -0.7 | 0.6 | 0.914 | 0.1 | -0.5 | 0.7 | 0.759 |
| Some college education^1,2^ | -0.2 | -0.8 | 0.4 | 0.534 | -0.1 | -0.6 | 0.5 | 0.845 |
| Annual income^3^ | -0.3 | -0.5 | 0.0 | 0.024 | -0.4 | -0.6 | -0.2 | 0.001 |
| Low vs high PLA | -1.3 | -1.8 | -0.9 | <.001 | -1.0 | -1.4 | -0.5 | <.001 |
| FI vs FS/NI vs NS | 1.7 | 1.2 | 2.2 | <.001 | 1.7 | 1.3 | 2.2 | <.001 |
| CI, confidence interval; FI, food insecurity; FS, food security; NI, nutrition insecurity; | | | | | | | | |
| NS, nutrition security; PLA, perceived limited availability. | | | | | | | | |
| ^1^ Reference category is ≥ Bachelor's degree. | | | | | | | | |
| ^2^ Includes Associate degree, trade school, or professional certificate. | | | | | | | | |
| ^3^ Annual income is standardized (1 standard deviation = 12,043 United States dollars). | | | | | | | | |
